# Supplementary material for: Modeling glioblastoma heterogeneity as a dynamic network of cell states
Source: Mol Syst Biol. 2021 Sep 16;17(9):e10105. doi: 10.15252/msb.202010105 (PMC8444284; doi:10.15252/msb.202010105)
Supplement: Supplementary file 6 — Source Data for Figure 5 [file MSB-17-e10105-s004.zip › Figure5A_sourcedata/GSEA_3071/hallmarks_stateVI.GseaPreranked.1623742557031/HALLMARK_XENOBIOTIC_METABOLISM.html]

Details for gene set HALLMARK\_XENOBIOTIC\_METABOLISM[GSEA]

|  || Dataset | state63071 |
| Phenotype | NoPhenotypeAvailable |
| Upregulated in class | na\_pos |
| GeneSet | HALLMARK\_XENOBIOTIC\_METABOLISM |
| Enrichment Score (ES) | 0.24755308 |
| Normalized Enrichment Score (NES) | 1.4072266 |
| Nominal p-value | 0.07174888 |
| FDR q-value | 0.12651172 |
| FWER p-Value | 0.515 |
Table: GSEA Results Summary

  

Fig 1: Enrichment plot: HALLMARK\_XENOBIOTIC\_METABOLISM      
 Profile of the Running ES Score & Positions of GeneSet Members on the Rank Ordered List

  

| PROBE | GENE SYMBOL | GENE\_TITLE | RANK IN GENE LIST | RANK METRIC SCORE | RUNNING ES | CORE ENRICHMENT || 1 | SERPINE1 |  |  | 0 | 1.673 | 0.0838 | Yes |
| 2 | ETS2 |  |  | 52 | 0.864 | 0.1129 | Yes |
| 3 | MT2A |  |  | 58 | 0.859 | 0.1546 | Yes |
| 4 | AOX1 |  |  | 182 | 0.619 | 0.1514 | Yes |
| 5 | SMOX |  |  | 196 | 0.604 | 0.1781 | Yes |
| 6 | UPP1 |  |  | 218 | 0.590 | 0.2018 | Yes |
| 7 | MAN1A1 |  |  | 250 | 0.563 | 0.2214 | Yes |
| 8 | SERTAD1 |  |  | 380 | 0.496 | 0.2105 | Yes |
| 9 | ASL |  |  | 421 | 0.482 | 0.2235 | Yes |
| 10 | SSR3 |  |  | 422 | 0.480 | 0.2476 | Yes |
| 11 | IGFBP4 |  |  | 571 | 0.433 | 0.2281 | No |
| 12 | PAPSS2 |  |  | 600 | 0.425 | 0.2417 | No |
| 13 | PTGR1 |  |  | 661 | 0.409 | 0.2455 | No |
| 14 | BCAT1 |  |  | 791 | 0.378 | 0.2286 | No |
| 15 | GSTO1 |  |  | 871 | 0.365 | 0.2250 | No |
| 16 | ACP1 |  |  | 970 | 0.350 | 0.2153 | No |
| 17 | PDLIM5 |  |  | 1062 | 0.335 | 0.2068 | No |
| 18 | FAH |  |  | 1082 | 0.333 | 0.2182 | No |
| 19 | SLC1A5 |  |  | 1169 | 0.320 | 0.2104 | No |
| 20 | BCAR1 |  |  | 1319 | 0.301 | 0.1841 | No |
| 21 | NFS1 |  |  | 1407 | 0.291 | 0.1745 | No |
| 22 | UGDH |  |  | 1548 | 0.274 | 0.1494 | No |
| 23 | GCNT2 |  |  | 1598 | 0.269 | 0.1493 | No |
| 24 | MAOA |  |  | 1659 | 0.263 | 0.1458 | No |
| 25 | XDH |  |  | 1780 | 0.253 | 0.1251 | No |
| 26 | PGRMC1 |  |  | 1889 | -0.262 | 0.1083 | No |
| 27 | CASP6 |  |  | 1996 | -0.278 | 0.0927 | No |
| 28 | ARPP19 |  |  | 2261 | -0.325 | 0.0357 | No |
| 29 | EPHX1 |  |  | 2327 | -0.339 | 0.0346 | No |
| 30 | IDH1 |  |  | 2526 | -0.384 | -0.0011 | No |
| 31 | CYFIP2 |  |  | 2619 | -0.404 | -0.0064 | No |
| 32 | PROS1 |  |  | 2647 | -0.411 | 0.0067 | No |
| 33 | ID2 |  |  | 3026 | -0.554 | -0.0705 | No |
| 34 | GAD1 |  |  | 3266 | -0.726 | -0.1005 | No |
| 35 | TGFB2 |  |  | 3340 | -0.804 | -0.0805 | No |
| 36 | HES6 |  |  | 3384 | -0.864 | -0.0492 | No |
| 37 | NINJ1 |  |  | 3500 | -1.117 | -0.0252 | No |
| 38 | FBLN1 |  |  | 3539 | -1.262 | 0.0275 | No |
Table: GSEA details [plain text format]

  

Fig 2: HALLMARK\_XENOBIOTIC\_METABOLISM: Random ES distribution      
 Gene set null distribution of ES for **HALLMARK\_XENOBIOTIC\_METABOLISM**
